# Uncovering drivers of dose-dependence and individual variation in malaria infection outcomes

**DOI:** 10.1371/journal.pcbi.1008211

**Published:** 2020-10-08

**Authors:** Tsukushi Kamiya, Megan A. Greischar, David S. Schneider, Nicole Mideo

**Affiliations:** 1 Department of Ecology & Evolutionary Biology, University of Toronto, Toronto, ON M5S 3B2, Canada; 2 Department of Ecology Evolutionary Biology, Cornell University, United States of America; 3 Program in Immunology, Stanford University, Stanford, California, United States of America; 4 Department of Microbiology and Immunology, Stanford University, Stanford, California, United States of America; UNITED STATES

## Abstract

To understand why some hosts get sicker than others from the same type of infection, it is essential to explain how key processes, such as host responses to infection and parasite growth, are influenced by various biotic and abiotic factors. In many disease systems, the initial infection dose impacts host morbidity and mortality. To explore drivers of dose-dependence and individual variation in infection outcomes, we devised a mathematical model of malaria infection that allowed host and parasite traits to be linear functions (reaction norms) of the initial dose. We fitted the model, using a hierarchical Bayesian approach, to experimental time-series data of acute *Plasmodium chabaudi* infection across doses spanning seven orders of magnitude. We found evidence for both dose-dependent facilitation and debilitation of host responses. Most importantly, increasing dose reduced the strength of activation of indiscriminate host clearance of red blood cells while increasing the half-life of that response, leading to the maximal response at an intermediate dose. We also explored the causes of diverse infection outcomes across replicate mice receiving the same dose. Besides random noise in the injected dose, we found variation in peak parasite load was due to unobserved individual variation in host responses to clear infected cells. Individual variation in anaemia was likely driven by random variation in parasite burst size, which is linked to the rate of host cells lost to malaria infection. General host vigour in the absence of infection was also correlated with host health during malaria infection. Our work demonstrates that the reaction norm approach provides a useful quantitative framework for examining the impact of a continuous external factor on within-host infection processes.

## Author summary

Infection outcomes are highly variable. Underlying this variation are many sources of heterogeneity with known quantitative impacts on outcomes, but elusive causal mechanisms. Focusing on the initial infection dose—which varies and is known to impact the outcome of infection in many disease systems—we explored how a biotic factor mediates host responses to infection and parasite growth. We fitted a dynamical model of within-host malaria infection to rodent experimental data spanning seven orders of magnitude of doses, using a Bayesian approach. Our results demonstrate that infection dose is a double-edged sword for the host response: when it is too high, responses can be activated less strongly; when it is too low, some responses to infection may be short-lived. We also uncovered sources of individual variation—usually considered experimental noise—that may represent subtle trait variation among hosts and parasites, and potentially new therapeutic targets. Our work highlights the value of combining a dynamical within-host infection model, with a hierarchical Bayesian approach, to interrogate the causal underpinnings of experimental observations.

## Introduction

Infections produce divergent outcomes. In human malaria, for example, outcomes of infection with the same parasite, *Plasmodium falciparum*, range from sub-clinical to fatal [1]. Understanding drivers of variation in infection outcomes is central to explaining why some hosts get sicker than others. Some host and parasite factors underlying this variation have well-understood mechanisms. For example, heterozygosity in the haemoglobin coding gene (i.e., sickle-cell trait, or HbAS) confers partial protection against falciparum malaria: sickle-cell trait individuals experience lower parasite load and reduced likelihood of life-threatening cerebral malaria and severe anaemia [2]. The resistance mechanism of this single locus trait has been corroborated by four decades of research demonstrating that sickling enhances clearance of infected red blood cells (iRBCs) by host immune effectors like macrophages [2]. However, unlike the sickle-cell trait, there are numerous sources of heterogeneity—including in the initial infection dose, nutrition, coinfection, and other genetic factors—for which quantitative impacts on outcomes have been observed, but causal mechanisms have remained elusive [3–6]. To establish a causal link between complex factors and infection outcomes, a key challenge is to quantify how a factor of interest mediates key host and parasite processes, such as host responses to infection and parasite growth [7].

In many disease systems, the initial infection dose is a key biotic factor that varies widely across infection events [8, 9]. Experimental infections in diverse systems show that increasing infection dose negatively impacts host fitness through reduced host vigour, survival and fecundity [10–17], which is likely linked to variation in the within-host parasite dynamics due to dose-dependence in parasite growth and host immune responses [4, 12, 18–21]. Complex interactions between immune responses and the initial infection dose have been revealed by molecular immunology studies of viral and bacterial systems. For example, the expression of pro-inflammatory cytokines (i.e., signalling molecules) and immune cells, can decrease with infection dose [22, 23], presumably due to enhanced evasion and escalated damage of host immune machinery through an increased abundance of reactive oxygen species [24]. Conversely, it has been shown that higher doses trigger distinct, and sometimes more robust, activation of innate and adaptive immune pathways [25–28]. From an applied perspective, a better understanding of the immunogenic dose-response relationship is pertinent for optimising vaccine dosing to ensure improved safety and efficacy [21, 29–31]. However, because the functional output of immune activities (e.g., the rate of immune-mediated iRBC clearance) is difficult to measure directly, it remains an open question how the initial infection dose influences host responses overall.

**Fig 1:**
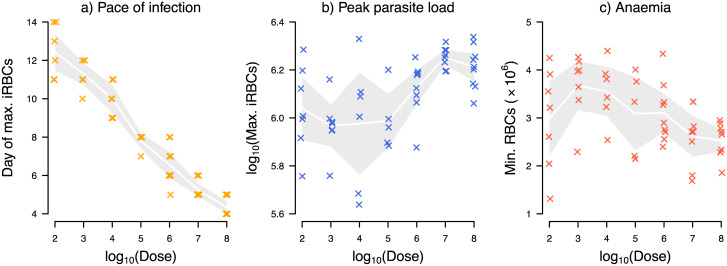
Higher initial infection doses increase a) the pace of infection (i.e., time until peak iRBC density), b) peak parasite load measured as the maximum iRBC count, and c) severity of anaemia during malaria infection measured as the minimum RBC count. There is also considerable variation in quantitative infection outcomes (i.e., parasite load and anaemia) within infection dose treatments. Data from Timms et al. [15] with 5 to 9 mice infected with the CW strain of *P. chabaudi*, at each dose. The crosses indicate data and the white lines and grey bands correspond to the means and 95% confidence intervals.

In malaria infections, the initial density of iRBCs at the start of blood-stage infection likely ranges in the order of hundreds to over millions [32–36], with greater numbers generally shown to increase mortality and worsen morbidity [13, 15]. Experimental manipulations of the initial infection dose, ranging from 100 to 100 million iRBCs, have demonstrated that larger doses increase the pace of infection with each order of magnitude reducing the time until peak infection by roughly 24 hours [15] (Fig 1a). Dose also impacts the peak parasite load: mice initially infected with 100 million parasites harbour roughly 60% more iRBCs at peak compared to those infected with 100 parasites [15] (Fig 1b). Furthermore, high doses induced more severe anaemia measured by the minimum red blood cell (RBC) count in mice on average [15] (Fig 1c). Previous mathematical modelling studies interpret these dose-dependent infection outcomes as a reflection of the underlying dose-dependence in host immune responses [4, 37].

While dose clearly influences malaria infection dynamics, these experimental data also reveal striking variability within dose treatments [15] (Fig 1), meaning that quantitatively diverse infection outcomes were observed across individuals receiving the same infection dose. This is despite the fact that hosts were inbred to homozygosity and parasites were also of single strain origin in the experiment [15]. Identifying the sources of such individual variation—usually considered experimental “noise”—may reveal biologically interesting, subtle trait variation among hosts and/or parasites, and thus new therapeutic targets (e.g., host responses to boost).

In the study of acute malaria infection, mathematical models have been fitted to the time course of experimental infections in mice to provide a quantitative understanding of parasite growth, pathogenesis and host responses to infection (e.g., [38–41]; see [42] for a review of earlier work). Infection triggers a variety of host responses, for example, indiscriminate clearance of RBCs, targeted clearance of iRBCs [39] and production of new RBCs to compensate for those lost to infection [39, 41]. It is well documented that these responses involve a complex cascade of interactions across multiple organisational scales from molecules, cells, and tissues to organ systems [43, 44]. However, it remains a challenge to scale up the details of finer level processes to an understanding of the net effect of host responses on parasite load and host health [45]. Data-driven mathematical modelling allows for the inference of these net effects, without necessarily requiring a detailed understanding of the underlying mechanisms.

Here, we fitted a dynamical model of within-host malaria infection to experimental data spanning seven orders of magnitude of initial doses, using a hierarchical Bayesian approach. By modelling the influence of dose on model parameters as a reaction norm, which describes the pattern of phenotypic expression of an organism across an environmental gradient, we identified drivers of the observed dose-dependent malaria parasite load and severity of malaria-induced anaemia. By explicitly modelling individual variation as model parameters, we also examined the origin of quantitatively diverse infection outcomes observed within single initial infection dose treatments.

## Methods

### System and experimental set-up

The rodent malaria system offers unique opportunities to investigate infection ecology, pathogenesis and host responses [46]. We examined previously published experimental data of C57BL/6 female mice infected with the CW strain of *Plasmodium chabaudi* [15]. In this experiment [15], infection was initiated with an intraperitoneal injection of iRBCs at seven different doses: 10^2^, 10^3^, 10^4^, 10^5^, 10^6^, 10^7^, 10^8^—and considerable variation in quantitative infection outcomes was observed both among and within dose treatments (Fig 1b & 1c). Details of the experiment are provided by Timms et al. [15] and data are available on Dryad [47].

### Model

#### Innate host responses to malaria infection

Hosts trigger a variety of responses to resist, tolerate and/or recover from infections. Here, we focused on two forms of rapid immune responses (on the order of minutes [48, 49]) that have been identified as the most pertinent to describing the acute blood-stage malaria infection [38–40].

The first response we modelled was general clearance of RBCs which may involve mechanisms such as retention of RBCs by the spleen and destruction of RBCs by immune effector cells [50, 51]. Clearing RBCs indiscriminately has been proposed as a host adaptation in the presence of malaria parasites to directly clear the parasites (i.e., top-down effect) as well as to limit the resource for the parasite (i.e., bottom-up effect) [52]. The second response we considered was the induction of innate immunity targeting iRBCs only, which is considered predominantly responsible for controlling the acute phase of malaria infection [43].

**Fig 2:**
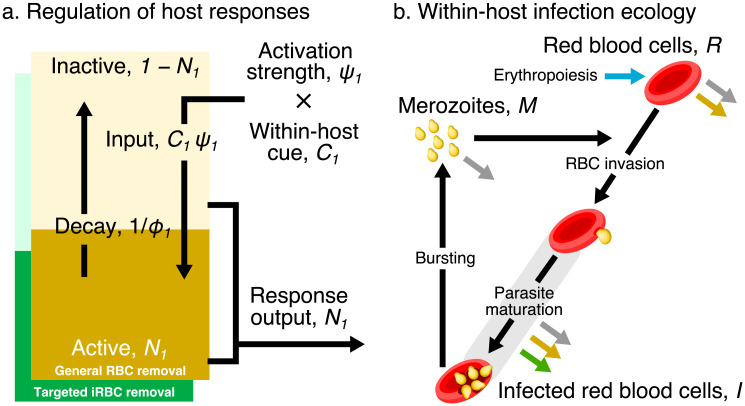
a) A dynamical regulation model of host responses against blood-stage malaria. We condensed the complexity of the vertebrate innate response against malaria into two independent pathways responsible for general RBC clearance and targeted iRBC clearance (represented by the yellow and green block, respectively). We modelled each pathway using a single differential equation, the activity of which is denoted *N*_*i*_ where the subscript *i* indicates the identity of each response: general RBC clearance, *i* = 1; and targeted iRBC clearance, *i* = 2. (The schematic shows the example of *i* = 1.) For each response type, the host detects a within-host cue, *C*_*i*_. The product of the cue and the strength constant, *ψ*_*i*_, activates the response. The activity of a response decays spontaneously with a half-life, *ϕ*_*i*_. The output of each host response feeds back to influence the within-host infection dynamics (indicated by the coloured arrows in panel b). b) Dynamics of RBCs and blood-stage malaria parasites within the host. Recruitment into and transitions among components of the asexual cycle are indicated with black arrows. Background mortality for different components is indicated by grey arrows. General clearance of RBCs and targeted clearance of iRBCs are marked with yellow and green arrows, respectively. Replenishment of RBCs (erythropoiesis) is indicated in blue.

We modelled regulation of host responses without delving into fine mechanistic details (i.e., we avoided mathematical descriptions of cytokine storms and subsequent cascades of effector responses). In part, this modelling choice was out of necessity because there is no complete map of innate immune responses against malaria [46]. Yet, it was also by design so that we would gain a functional understanding of host responses with minimal complexity. Biologically, responses modelled here may reflect the output of an entire module of proteins and signal transduction pathways that lead to the production of effector cells. Specifically, we used a single ordinary differential equation (ODE) to describe the change in the functional output of each response (Fig 2a; yellow and green block, respectively). We assumed that the maximum possible activity of each response is fixed, at one, and we tracked the dynamics of its proportional activity, *N*_*i*_, where *i* indicates the response identity (general RBC clearance, *i* = 1; targeted iRBC clearance *i* = 2)

$$\begin{array}{c} \frac{dN_{i}\left(t\right)}{dt}=\psi_{i}\;C_{i}\left(t\right)(1-N_{i}\left(t\right))-\frac{N_{i}\left(t\right)}{\phi_{i}}. \end{array}$$ (1)

We defined the activity of *N*_1_ and *N*_2_ as the proportion of RBCs and iRBCs cleared by indiscriminate and targeted mechanisms per day, respectively. We assumed that there is no response output in the absence of infection, i.e., *N*_1_(*t* = 0) = *N*_2_(*t* = 0) = 0, consequently assuming a stable RBC population and that there is no constitutive immune activity.

We modelled the signalling input that activates each response as a function of a within-host cue, *C*_*i*_(*t*) and a constant determining the strength of activation, *ψ*_*i*_ (Eq 1). Host innate immune responses against malaria are thought to be triggered by pathogen-associated molecular patterns (PAMPs) such as GPI anchors, haemozoin, parasite DNA and RNA [44, 46]. Assuming that the abundance of PAMPs reflects that of iRBCs, our model considers the relative density of iRBCs compared to its observed maximum in any infection across all treatment groups, as the within-host cue for general RBC clearance and targeted iRBC clearance, i.e., $C_{1}\left(t\right)=C_{2}\left(t\right)=\frac{I(t)}{\text{max}\;I}$ where *I*(*t*) and max *I* are the iRBC density at time *t* and the maximum observed iRBC density, respectively. The latter was reported at 2.18 × 10^6^ from this dataset [15]. We assumed that each response activity decays spontaneously with a half-life of *ϕ*_*i*_.

Our two-parameter approach—involving only an activation constant, *ψ*_*i*_, and activity half-life, *ϕ*_*i*_—to modelling each host response was inspired by Kochin et al. [38] who used a single ODE to model innate immunity against malaria parasites governed by density-dependent response activation and constant decay. However, we interpreted host response activity differently from their study: i.e., we modelled the proportion of RBCs and iRBCs cleared per day whereas they modelled the number of immune cells. Our study also extends the approach to the dynamics of general RBC clearance.

#### Within-host infection dynamics of blood-stage rodent malaria

We used a system of ODEs to model the blood-stage asexual cycle of *P. chabaudi*, tracking the density of uninfected RBCs (hereafter, uRBCs), *R*, iRBCs, *I*, and extracellular parasites called merozoites, *M*, in a microlitre of blood (Fig 2b). In this model, we assumed that RBCs are constantly replenished to maintain a homeostatic equilibrium, thus the daily rate of erythropoiesis in the absence of infection is defined as *R*_*c*_ *μ*_*R*_, where *μ*_*R*_ is the baseline RBC mortality rate. We estimated *R*_*c*_ at 8.89 × 10^6^ from data [15] as the average RBC density of 10 uninfected mice between Day 7 and 14 during which time the RBC density appears stable. During the acute phase of malaria infection, the host upregulates erythropoiesis to restore RBCs lost to malaria-induced anaemia [53, 54]. Following a previous study [39], we modelled this upregulation as a product of the deviation from the homeostatic equilibrium, *R*_*c*_ − *R*(*t*) where *R*(*t*) is the RBC density at time *t*, and the proportion of the deviation from the homeostatic equilibrium restored by the host per day, *ρ*.

Given that *N*_1_ and *N*_2_ were defined as the proportion of cells cleared by indiscriminate and targeted mechanisms per day, respectively, it was convenient to convert the proportions into daily rates at which cells are cleared in the dynamical within-host model. To do this, we equate $N_{i}=1-e^{-X_{i}}$, where *X* is the daily rate of clearance. Solving for *X*, we obtained the rate of general RBC clearance as −*ln*(1 − *N*_1_) and the rate of targeted iRBC clearance as −*ln*(1 − *N*_2_). Therefore, the sum of the baseline rate, *μ*_*R*_ and −*ln*(1 − *N*_1_) constitutes the daily mortality rate of uRBCs. uRBCs then become infected at a rate proportional to the density, *M*, and invasion rate, *p*, of merozoites. Together the dynamics of uRBCs is expressed as:

$$\begin{array}{ccc} \frac{dR(t)}{dt} & = & R_{c}\;\mu_{R}\;+\;\rho \left(R_{c}-R\left(t\right)\right)-\;\left(\mu_{R}-ln\left(1-N_{1}\right)\right)\;R\left(t\right)-p\;R\left(t\right)\;M\left(t\right). \end{array}$$ (2)

Following merozoite invasion, iRBCs remain subjected to background mortality, *μ*_*R*_ and general RBC clearance, −*ln*(1 − *N*_1_). In addition, infected cells are cleared by targeted immunity at a rate −*ln*(1 − *N*_2_): here, we note that estimates of −*ln*(1 − *N*_2_) may be slightly inflated by iRBCs that commit to sexual reproduction (usually less than 2% of iRBCs [55]) because our model does not consider *Plasmodium* sexual reproduction. We modelled the development of iRBCs using a gamma-chain trick (also known as linear chain trick) [56, 57], which consists of a series of ODEs:

$$\begin{array}{ccc} \frac{dI_{1}\left(t\right)}{dt} & = & p\;R\left(t\right)M\left(t\right)-\left(\mu_{R}-ln\left(1-N_{1}\right)-ln\left(1-N_{2}\right)+\tau \right)I_{1}\left(t\right) \end{array}$$ (3)

$$\begin{array}{ccc} \frac{dI_{i}\left(t\right)}{dt} & = & \tau \;I_{i-1}\left(t\right)-\left(\mu_{R}-ln\left(1-N_{1}\right)-ln\left(1-N_{2}\right)+\tau \right)I_{i}\left(t\right)\;\;\text{for}\;2\leq i\leq n \end{array}$$ (4)

where τ=nα, and *α* is the average cell cycle duration, which is 24 hours for *P. chabaudi* [58]. The number of compartments in the series, *n*, reflects the assumption about the variance in the developmental time, which is inversely proportional to *n* [56]. At one compartment per cycle (i.e., *n* = 1, e.g., [59, 60]), the assumption is that the developmental time is exponentially distributed, with large variance, i.e., *α*^2^ [61]. The variance decreases with the number of compartments, and the variance tends to disappear as the number of compartments approaches infinity (i.e., *n* → ∞)—a scenario equivalent to discrete-time models (e.g., [62, 63]) and delay-differential equation models [64] that assume there is no variation in the developmental period [56]. It has been shown that models with few compartments tend to estimate greater asexual multiplication, compared to discrete-time models [61, 65]. With enough compartments (e.g., one compartment per hour of parasite development [56]), however, the outcome of a gamma-chain ODE model converges to the discrete-time model [65]. We arrived at the choice of *n* = 12 (one compartment every two hours) for computational efficiency, and because our preliminary analysis showed that the infection dynamics were quantitatively comparable to that of a 24 compartment model (one compartment per hour).

Finally, the production of merozoites is determined by parasite burst size (i.e., the number of progeny parasites emerging from an iRBC), *β*, and the number of bursting cells, *τ I*_*n*_(*t*). Merozoites are lost as they invade new RBCs and through background mortality, *μ*_*M*_. We ignored immune-mediated clearance of merozoites because its effect on the parasite dynamics is functionally similar to clearing iRBCs and comparatively less important for describing the acute phase of malaria infection than general RBC clearance or targeted iRBC clearance [4, 39],

$$\begin{array}{ccc} \frac{dM(t)}{dt} & = & \beta \;\tau \;I_{n}\left(t\right)-p\;R\left(t\right)M\left(t\right)-\mu_{M}\;M\left(t\right). \end{array}$$ (5)

#### Initial conditions

We set the initial RBC density, *R*(*t* = 0), to the values reported per mouse by Timms et al. [15]. In the experiment [15], malaria infection was initiated with an intraperitoneal injection to mimic the initial cohort of blood-stage malaria parasites following release from the liver. Assuming that the initial parasite growth is near-exponential (for the first three records of iRBCs per mouse), we estimated the initial infection dose per *μl* of blood in each mouse as the intercept of a linear regression model with the natural logarithm of iRBCs as the response and the time since infection as a predictor. At a preliminary phase, we also estimated the initial infection dose simultaneously with the rest of the model parameters. Because these two methods for estimating initial dose yielded analogous results (see S1a Appendix) and the regression method allowed us to estimate one fewer parameter in the main Bayesian parameter inference procedure (described below), we present results based on the initial infection dose estimated by the regression method. Finally, we defined the age structure of the inoculated iRBCs to schedule the bursting of iRBCs in the initial cohort. Assuming that all inoculated iRBCs commit to producing merozoites (i.e., ignoring the possibility that some of them produce transmission stages instead), the timing of bursting can be described using a symmetrical beta distribution [64]. We assumed a moderately synchronised blood-stage cycle (i.e., with the shape parameter, *s* = 10). Our preliminary exploration indicated that the daily RBC and iRBC measurements were insensitive to the *s* parameter. We discretised the beta distribution into 12 compartments (for Eqs 3 and 4) by dividing the cumulative density function into 12 intervals.

### Hierarchical Bayesian inference

Bayesian causal inference is an effective tool to paint a picture of processes that generated data [67]. We fitted our ODE model describing the dynamics of RBCs (Eq 2) and iRBCs (Eqs 3 and 4) to the corresponding time-series data from 51 mice [15] using a Bayesian statistical approach. Statistical inference in a Bayesian framework incorporates prior knowledge and uncertainty about model parameters and updates the belief about them (by computing the posterior probability of parameters) based on observations (through a likelihood function) [68]. Besides its conceptual merits [68, 69], Bayesian inference has a pragmatic appeal for its ability to estimate parameters in high dimensional spaces, for example, in hierarchical models, which are used when observations are organised in multiple levels of sampling units [70, 71]. In this study, we considered two levels of sampling units: treatments (i.e., infection doses) and subjects (i.e., individual mice).

#### Dose and individual-specific effects

For the dynamical model (Eqs 1 to 5), we estimated dose- and individual-specific effects in a set of seven parameters including the response activation strength of host responses *N*_1_ and *N*_2_ (*ψ*_1_ and *ψ*_2_, respectively), half-life of those responses (*ϕ*_1_ and *ϕ*_2_, respectively), erythropoiesis upregulation (*ρ*) and parasite burst size (*β*). Below, we collectively refer to the parameter set as *θ* (*θ* ∋ *ψ*_1_, *ψ*_2_, *ϕ*_1_, *ϕ*_2_, *ρ*, *β*) and refer to a parameter in the set using an index, *k*. The prior distributions for these parameters are provided in Table 1 and further detailed in S1b Appendix.

**Table 1:** Descriptions of model parameters and their fixed values, or prior distributions used in Bayesian statistical inference. Estimated parameters are indicated by an asterisk on the description. We assigned a generic, weakly informative prior, except for erythropoiesis upregulation, *ρ*, burst size, *β*, and standard deviations of *log*_10_ RBC and iRBC density, *σ*_*RBC*_ and *σ*_*iRBC*_, for which there exist specific prior information from previous studies. Further details of the priors and comparisons with estimated posterior probability densities, and prior sensitivity analyses are provided in S1b Appendix.

Symbol	Description	Fixed value or prior distributions	[Source]
Host responses			
*ψ*_*i*_	*Activation strength of *N*_*i*_	$\text{exp}(N\left(ln\left(1\right)+5,\sqrt{5}\right)$	
*ϕ*_*i*_	*Half-life of *N*_*i*_	$\text{exp}(N\left(ln\left(1\right)+5,\sqrt{5}\right)$ day	
Within-host infection dynamics			
*R*_*c*_	RBC density at homeostatic equilibrium	8.89 × 10^6^ per *μl*	[15]
*maxI*	Maximum iRBC density observed in Timms et al. [15]	2.18 × 10^6^ per *μl*	[15]
*μ*_*R*_	Baseline RBC mortality rate	0.025 per day	
*ρ*	*Proportion of deviation from *R*_*c*_ restored per day	0.25×exp(N(0,0.25))	[39]
*β*	*Parasite burst size	7×exp(N(0,0.25))	[39]
*p*	Merozoite invasion rate	8 × 10^−6^ per day	[39]
*α*	*P. chabaudi* RBC cycle duration	1 day	[58]
*n*	Number of RBC cycle components	12	
*s*	Degree of synchronous bursting	10	
*μ*_*M*_	Merozoite mortality rate	48 per day	[66]
Reaction norms and individual variation			
*δ*_*p*_	*Dose-dependent reaction norm slope for parameter *p*	N(0,2.5)	
*σ*_*u*,*p*_	*Individual deviation for intercept	N(0,1)	
*σ*_*v*,*p*_	*Individual deviation for slope	N(0,1)	
Measurement errors			
*σ*_*RBC*_	*Standard deviations for total RBC density	$N(5\times 10^{5},5\times 10^{5}/10)$	[39]
*σ*_*iRBC*_	*Standard deviations for *log*_10_ iRBC count	N(0.13,0.13/10)	[39]

Instead of modelling each dose treatment as a discrete group (or a character state), we consider the entire range of the initial infection dose as an environmental gradient. In other words, we modelled each parameter of the dynamical model, *θ*_*k*_, as a reaction norm, which describes the pattern of phenotypic expression of an organism across an environmental gradient [72, 73]. The simplest and most commonly used reaction norm assumes a linear relationship between the environment and phenotype expression and consists of two components: the mean intercept, i.e., the phenotype expressed against the “average” environment, and the slope, i.e., the degree of phenotypic change along with the environment. We estimated the mean intercept, ${\hat{\theta }}_{k}$ (dose was centred so that the intercept is at the middle dose of 10^5^) and the mean slope of the reaction norms, ${\hat{\delta }}_{k}$.

Within each dose treatment, Timms et al’s dataset [15] contains repeated measures from replicate mice that showed marked individual variability (Fig 1b & 1c). We sought to identify the source of this variability by explicitly modelling individual variation in *θ*_*k*_ among mice through partial pooling. This means that a given parameter was considered a sample from a common population distribution with a mean—in this case the intercept, ${\hat{\theta }}_{k}$, and the slope, ${\hat{\delta }}_{k}$—and the deviation of the parameter from the mean for each individual, *i*, which we express as *u*_*k*,*i*_ and *v*_*k*,*i*_ for the intercept and slope variation, respectively. We assumed that the individual deviations, *u*_*k*,*i*_ and *v*_*k*,*i*_, are samples of a normal distribution with standard deviations, *σ*_*u*,*k*_ and *σ*_*v*,*k*_ that we estimated from data [74].

Together, dose- and individual-specific parameter, *θ*_*k*,*i*_, is expressed as:

$$\begin{array}{c} \theta_{k,i}={\hat{\theta }}_{k}+u_{k,i}+\left({\hat{\delta }}_{k}+v_{k,i}\right)\times Dose_{i} \end{array}$$ (6)

where *Dose*_*i*_ indicates the dose treatment applied to individual *i*. *Dose* was coded as {−3, −2, −1, 0, 1, 2, 3} such that the intercept was centred at the initial infection dose of 10^5^. As is customary in quantitative genetics [72] and hierarchical modelling [75], we modelled a covariance structure describing the association among parameters in individual deviation following [75].

#### Likelihood

A Bayesian approach requires a likelihood function to assess the probability of observing the data given model parameters and associated predictions. Our log-likelihood function assumed that the measurement error for the total density of RBCs (i.e., sum of uRBCs and iRBCs), and iRBCs is distributed normally and *log*_10_-normally, respectively [40, 76]:

$$\begin{array}{ccc} \text{ln}L & = & \sum_{i}^{n_{\text{mice}}}\{\sum_{t}^{n_{\text{time}}}\;\text{ln}\{\frac{1}{\sigma_{\text{RBC}}\sqrt{2\pi }}\text{exp}[-\frac{{\left(D_{i,t}^{\text{RBC}}-M_{i,t}^{\text{RBC}}\right)}^{2}}{2{\left(\sigma_{\text{RBC}}\right)}^{2}}]\}\\ & + & \sum_{t}^{n_{\text{time}}}\;\text{ln}\{\frac{1}{\sigma_{\text{iRBC}}\sqrt{2\pi }}\text{exp}[-\frac{{\left({\text{log}}_{10}\left(D_{i,t}^{\text{iRBC}}+1\right)-{\text{log}}_{10}\left(M_{i,t}^{\text{iRBC}}+1\right)\right)}^{2}}{2{\left(\sigma_{\text{iRBC}}\right)}^{2}}]\}\} \end{array}$$ (7)

where $D_{i,t}^{\text{RBC}}$ and $D_{i,t}^{\text{iRBC}}$ are the observed count of total RBCs and iRBCs, $M_{i,t}^{\text{RBC}}$ and $M_{i,t}^{\text{iRBC}}$ are the model predictions of total RBCs and iRBCs for individual *i* at time *t*. We estimated standard deviations, *σ*_RBC_ and *σ*_iRBC_ for the total RBC and iRBC count, respectively, with specific informative priors based on [76] (Table 1).

Our modelling focused on the first wave of infection before iRBCs recrudesce and adaptive immunity starts to take effect [39]. Thus, *n*_time_ was defined {16, 16, 13, 11, 11, 10, 8} for the seven dose treatments, respectively, noting that higher doses lead to shorter time series because of the faster pace of infection (Fig 1a). We further subsetted the dataset by removing instances of atypical dynamics (S1c Appendix for more details). In total, we fitted data from 51 individual mice (*n*_mice_ = 51).

#### MCMC sampling

Estimating the posterior probability density of parameters of a complex model requires a Markov Chain Monte Carlo (MCMC) sampling algorithm, which can be computationally intensive for large hierarchical models. Our model was written in Stan 2.18.2 and fitted through the RStan interface [77, 78], which provides an efficient general-purpose MCMC sampler (No-U-Turn Hamiltonian Monte Carlo) and a Bayesian inference environment. The model was fitted in parallel in four independent chains, each with 4000 sampled iterations and 1000 warmup iterations. For diagnostics, we confirmed over 400 effective samples and ensured convergence of independent chains using the $\hat{R}$ metric (values below 1.1 are considered an indication of multi-chain convergence) for all parameters [68, 79]. The computer programmes used in the present study are available in the S1d Appendix.

## Results and discussion

**Fig 3:**
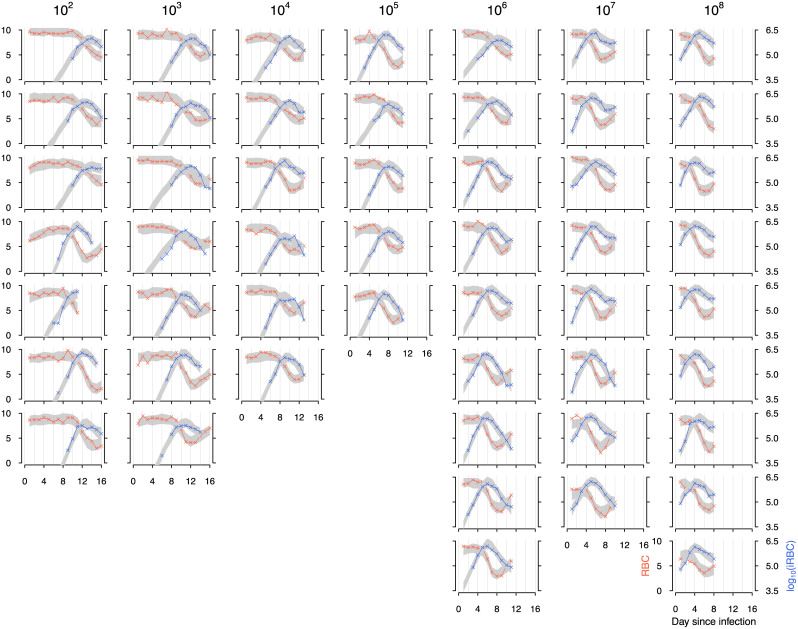
The fit of the full model (with parameters defined by Eq 6) to the density of RBCs (orange) and iRBCs (blue) for individual mice inoculated at 10^2^, 10^3^, 10^4^, 10^5^, 10^6^, 10^7^ and 10^8^ infected cells. Each column corresponds to the initial infection dose given at the top. The crosses indicate data and grey bands correspond to 95% predictive intervals of the full model, incorporating uncertainty in parameter estimation and sampling.

Our fitted model accurately describes the daily time course of RBCs and iRBCs during the acute phase of malaria infection in mice, initiated at doses spanning seven orders of magnitude, from 10^2^ to 10^8^ iRBCs (Fig 3, S1e Appendix). We found evidence for dose-dependence in key parameters of host responses underlying the dynamics of RBCs and iRBCs. Additionally, even under the highly controlled condition of Timms et al.’s experiment [15]—with the single strain combination of hosts (C57/BL6) and *P. chabaudi* parasites (CW)—we identified individual variation in host and parasite traits that influences the variation in infection outcomes independent of the dose treatment. Below, we closely examine the sources and impacts of dose-dependence and individual variation in different parameters of infection and initial conditions.

### Dependence on the initial infection dose

**Fig 4:**
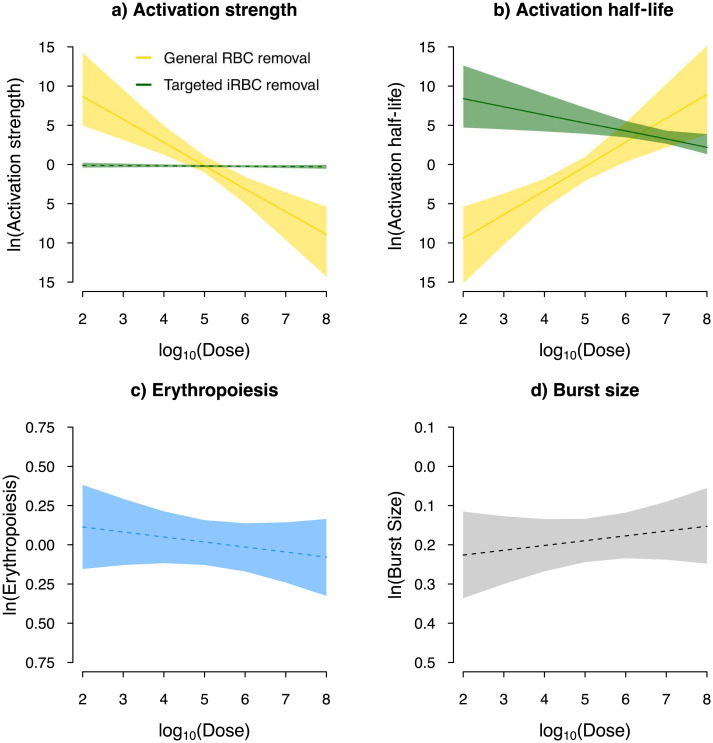
Dose dependence in some parameters of host responses, but not parasite traits. Plotted are the estimated relationship between the initial infection dose and a) the activation strength (*ψ*_1_ and *ψ*_2_), b) activity half-life (*ϕ*_1_ and *ϕ*_2_) of general RBC clearance (yellow) and targeted iRBC clearance (green), c) erythropoiesis upregulation, *ρ*, and d) parasite burst size, *β*. The line and band indicates the median prediction and 95% predictive intervals, respectively. The solid line indicates a statistically significant sign of dose-dependence (See S1b Appendix for the prior and posterior distributions).

**Fig 5:**
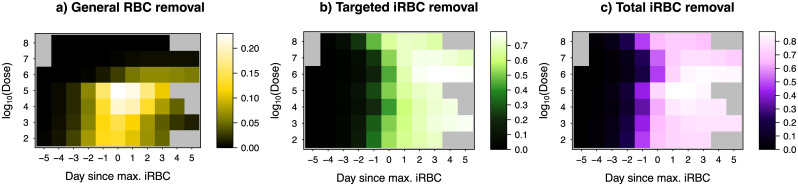
Host responses against malaria depend on the initial infection dose. Shown are the median predicted host responses (colour) as a function of time centred around the dose-dependent day of peak infection (x-axis) and the initial infection dose (y-axis) for a) general RBC clearance (i.e., proportion of RBCs, indiscriminately of their infection status, cleared by the host per day), b) targeted iRBC clearance (i.e., proportion of iRBCs cleared by the host per day), c) the total iRBC clearance (the sum of a and b). The grey region indicates that the model was not fitted for the day because either data do not exist or the day is beyond the first wave of infection.

We found evidence that increasing dose had two opposing effects on general RBC clearance. First, increasing dose reduces the activation strength of this response (Fig 4a). The lower peak responses estimated for high doses (Fig 5a) were attributable to a negative influence of the initial dose on the response activation strength (Fig 4a), supporting the notion that higher infection doses enhance the parasites’ ability to evade/suppress host responses and/or damage host machinery [24]. In contrast, we found that dose facilitates the same response by inducing a longer action (Fig 4b). The positive relationship between dose and activity half-life of these responses (Fig 4b) explained the relatively low peak response at low doses (Fig 5a). As a result of these two opposing effects of dose, the strongest host response in general RBC clearance was predicted at an intermediate dose, 10^5^ (white squares in Fig 5a). This finding draws a comparison to another data-driving modelling work demonstrating that the maximum immune protection against influenza is generally achieved at an intermediate dose due to an interplay between innate and adaptive responses [21]. Further studies in other disease systems are desired to probe the generality of the intermediate peak and understand diverse mechanisms that may generate this pattern.

Following the peak infection day, malaria parasites find themselves in a hostile within-host environment as over 70% of the iRBC population per day is cleared by host immunity targeting iRBCs (Fig 5b). Combining targeted and indiscriminate responses, up to 80% of iRBCs were cleared per day by the host one to three days after the peak of infection (Fig 5a). The activation of targeted iRBC clearance was estimated to be independent of the initial infection dose (Fig 4a) while we found that the half-life of this response decreased with infection dose (Fig 4b). This result is consistent with the faster waning of parasite clearance with dose observed by Metcalf *et al*. [37] who speculate mechanisms including enhanced antigen escape, reduced immune memory due to low RBC availability, depleted immune effectors and downregulation by the host or parasites. Without this dose-dependent effect, our sensitivity analysis demonstrated that the peak targeted iRBC clearance would occur at high doses, where higher iRBC density triggers an elevated response (S1f Appendix).

**Fig 6:**
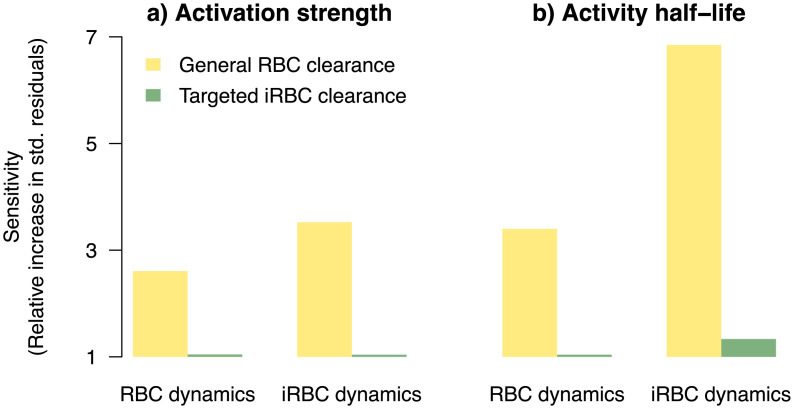
RBC and iRBC dynamics are most sensitive to dose-dependence in general RBC clearance. Plotted is the sensitivity of RBC and iRBC dynamics to dose-dependence of a) activation strength and b) activity half-life in general RBC clearance and targeted iRBC clearance (green), calculated as the sum of absolute standardised residuals (see S1e Appendix for details) relative to the full model (with parameters defined by Eq 6).

To assess the relative importance of dose-dependence in the host responses, we examined the sensitivity of the model fit to whether host response parameters were dose-dependent (by setting $\theta_{k,i}={\hat{\theta }}_{k}+u_{k,i}$, where *k* = {*ψ*_1_, *ϕ*_1_}, *k* = {*ψ*_2_, *ϕ*_2_}, for the two responses, respectively). We found that the dynamics of RBCs were most sensitive to dose-dependence in general RBC clearance: goodness of model fit declined by 2.6-fold and 3.4-fold, respectively, when dose-dependence in activation strength or activity half-life was ignored (Fig 6). Interestingly, even though the targeted response clears more iRBCs than the general response (Fig 5a & 5b), we found that iRBC dynamics were also overwhelmingly more sensitive to dose-dependence in general RBC clearance (Fig 6): goodness of model fit declined by 3.6-fold and 7-fold, respectively, when dose-dependence in either activation strength or activity half-life (Fig 6) was ignored. While the mechanism through which the host clears RBCs indiscriminately remains an open question, the functional importance of general clearance of RBCs is apparent from our work here and the work of others [41]. Furthermore, the distinct patterns of dose-dependence we found (Fig 4a & 4b) suggest that there is separate host machinery for specifically clearing iRBCs and clearing RBCs indiscriminately.

Previously, dose-dependence in infection dynamics has been attributed to ineffective clearance of iRBCs at high doses due to handling time in innate immune effectors targeting iRBCs [37]. Our findings offer an alternative explanation that dose promotes some aspects of some response while debilitating others. Of largest effects, we found that dose increases the half-life, but reduces activation strength, of general RBC clearance (Figs 4 & 6). This finding likely reflects complex mechanisms through which the initial infection dose impacts different aspects of host response machinery [24–28, 80]. Regardless of the mode of defence involved (i.e., indiscriminate or targeted iRBC clearance) and mechanisms (i.e., handling time, damage to host machinery, or immune evasion), the two distinct modelling frameworks demonstrate that malaria parasites are at an advantage at high initial infection dose due to less efficient host responses to clear iRBCs. We did not find significant evidence that upregulation in erythropoiesis or parasite burst size depends on the initial iRBC density (Fig 4c & 4d). Therefore, our findings suggest that dose-dependent variation in infection dynamics observed across initial infection dose treatments in Timms et al. [15] was driven by the interaction between the initial infection dose and host responses, but not plasticity in malaria parasites injected at different doses as predicted by Mideo *et al*. [76].

### Individual variation

**Fig 7:**
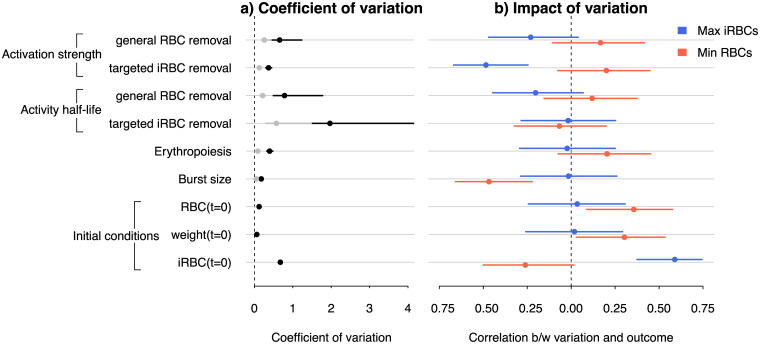
Individual variation in parameters of host responses and parasite growth impacts quantitative infection outcomes. a) Coefficient of variation among individuals in specific model parameters and initial conditions. Variation in the dose-independent (intercept) term, *σ*_*u*,*k*_ and dose-dependent (slope) term *σ*_*v*,*k*_. is indicated in black and grey, respectively. b) Correlation coefficients between individual-level estimates of parameters or initial conditions, and the infection outcome measured as the maximum and minimum densities of iRBCs (blue) and RBCs (red), respectively. The error bars indicate the 95% confidence interval. Statistical significance is indicated when the confidence interval does not intersect 0 (dashed line).

We explicitly modelled individual variation in each fitted parameter among the inbred mice using hyperparameters, *σ*_*u*,*k*_ and *σ*_*v*,*k*_. These sources of variation—analogous to subject-level random effects in regression analyses—capture unobserved heterogeneity among individuals in a sample, independent of the experimental manipulation in the initial infection dose. We found evidence of individual variation in every host and parasite parameter of the model describing malaria infection ecology (Fig 7a; S1b Appendix). There was no evidence of moderate or stronger correlation (*r* > 0.3) among individual variation in parameter values (S1g Appendix), suggesting that there are no clear trade-offs nor facilitation among different arms of host responses.

To understand the functional importance of individual variation in estimated model parameters, as well as random variation in the reported initial RBC density, host weight, and initial infection dose, we computed the correlation coefficient, *r*, between individual variation and two key infection outcomes: parasite load (maximum iRBC density) and anaemia severity (minimum RBC density). We identified two individual-level correlates of the maximum iRBC density (Fig 7b). First, we found that better suppression of the peak parasite load was associated with mice that activated the response to clear iRBCs more strongly than average through a targeted mechanism (Fig 7b). While it is not clear what underlies this within-strain variation, our finding, nonetheless, demonstrates the functional importance of non-genetic heterogeneity in immune responses, drawing parallels to invertebrate systems in which intrinsic within-clone variation (e.g., differences in sizes at birth and molecular mechanisms of immune responses) are thought to impact susceptibility to infection [81–83]. With the rodent malaria system, it may be possible to identify the mechanistic causal agents, for example, by characterising within-strain variation in immune effector expression prior to and throughout the course of infection. We also found that variation in the initial infection dose within dose treatments correlated positively with the peak parasite load (Fig 7b). In the context of Timms et al.’s [15] experiments, variation in the initial infection dose may be attributable, for example, to random experimental variability in sampling by a syringe and in the parasites’ ability to reach blood vessels following intraperitoneal injection. While neither of these sources of random variation are relevant outside the lab, the observation that higher doses—both among and within dose treatments—increase parasite burden [15] (Figs 1 & 7b) highlights the need to better understand any causes of variation in pre-blood-stage parasite densities in natural malaria infections, both within and among *Plasmodium* species [58]. One such source of variation is pre-blood-stage host immunity that develops in response to exposure to the parasite stage injected by mosquitoes (sporozoites) and has been shown effective in reducing the number of liver-stage parasites from which blood-stage merozoites originate [84].

There were also three significant individual-level correlates of anaemia severity (Fig 7b). First, we found a significant negative effect of burst size meaning that infections initiated with a parasite population that happens to proliferate and exhaust RBCs faster than average caused more severe anaemia (Fig 7b). In addition, mice that were heavier or had more RBCs before infection suffered less severe anaemia (Fig 7), suggesting that general host vigour in the absence of infection is an indicator of host health under malaria infection. Because these measurements of host vigour did not correlate with the peak parasite load, host vigour can be interpreted as an indicator of tolerance (i.e., a host’s ability to minimise anaemia), rather than a resistance (i.e., a host’s ability to minimise parasite burden) mechanism [85].

In general, we found that the magnitude of individual variation, estimated here as the coefficient of variation, did not coincide with whether or not a particular trait impacted an infection outcome. For example, we estimated large individual-level variation in the half-life of host response for targeted clearance of iRBCs (Fig 7a), yet infection outcomes were insensitive to this variation (Fig 7b). Conversely, we detected a small coefficient of variation in burst size (Fig 7a), but this small variation showed a marked impact on the severity of anaemia (Fig 7b). The misalignment between the magnitude and impact of individual variation poses a challenge from a clinical perspective because it is not clear whether those parameters can be estimated with sufficient precision from patient data. Finally, despite the fact that the dynamics of RBCs and iRBCs are ecologically coupled (i.e., malaria parasites are consumers of RBCs), we found that the impact of individual variation on anaemia severity was not coupled to that of parasite load. These findings indicate that resistance and tolerance are likely uncorrelated at the individual level in the rodent malaria system.

### Conclusion

We examined drivers of dose-dependent malaria parasite load and the severity of malaria-induced anaemia. We also shed light on the role of unobserved heterogeneity in producing diverse infection outcomes, identifying sources of subtle variation beneath an experimental treatment. More often than not, infection experiments are structured in multiple levels of sampling units (e.g., host, parasite genotypes, presence of coinfection, and drug treatments) with many replicates within treatment groups. Observations from such experiments contain multiple sources of variation whose effects on infection outcomes are difficult to disentangle directly. Our study demonstrates that the combination of dynamical within-host model and a Bayesian approach is a powerful tool for causal inference of infection outcome variation.

## Supporting information

S1 Appendix a) Estimation of the initial infection dose, b) Graphical summary of prior and posterior distributions, c) Mice excluded from model fitting, d) Computer programmes, e) Assessment of model fit: standardised residuals, f) Sensitivity of targeted iRBC clearance to dose-dependent half-life, and g) Correlations of individual variation.
(PDF)
