# Supplementary material for: Uncovering drivers of dose-dependence and individual variation in malaria infection outcomes
Source: PLoS Comput Biol. 2020 Oct 8;16(10):e1008211. doi: 10.1371/journal.pcbi.1008211 (PMC7544130; doi:10.1371/journal.pcbi.1008211)
Supplement: S1 Appendix — a) Estimation of the initial infection dose, b) Graphical summary of prior and posterior distributions, c) Mice excluded from model fitting, d) Computer programmes, e) Assessment of model fit: standardised residuals, f) Sensitivity of targeted iRBC clearance to dose-dependent half-life, and g) Correlations of individual variation. (PDF) [file pcbi.1008211.s001.pdf]

Supporting Information S1

a) Estimation of the initial infection dose in individual mice

We estimated the initial infection dose for each mouse using two methods. First, assuming that the initial parasite growth rate is near-exponential (for the first three days of observations), we estimated the initial infection dose per microliter of blood in each mouse as the intercept of a linear regression model with the natural logarithm of iRBCs as the response and the time since infection as a predictor. We extrapolated this estimate to the initial injected dose using the information about the individual mouse weight reported by Timms et al. [1] and assuming that there is 95 ml of blood per kg of a female mouse [2] (Fig. Aa; blue circles). Alternatively, we estimated the initial infection dose simultaneously with the rest of the model parameters using a Bayesian approach detailed in the main text (95% predictive band of which is shown in grey; Fig. Aa). We found that the two methods converge and both estimate higher than reported injected doses at low doses (Fig. Aa). Crucially, the rank order of the reported infection dose was preserved in our estimates (Fig. Aa).

We explored the possibility that the regression method was affected differently among dose treatments by dose-dependent iRBC clearance at an early stage of infection. We did not find evidence that the estimated slopes in the regression method were affected by the initial infection dose (Fig. Ab). Thus, it is unlikely that our estimates of the initial infection dose were biased differently among dose treatments by early host immunity.

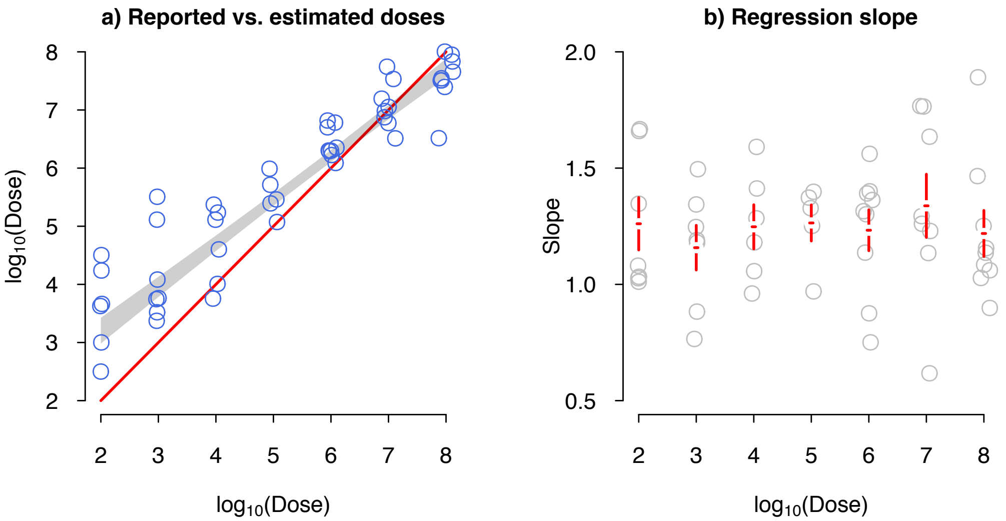

Figure A: a) The reported and estimated initial infection dose. The blue circles represent the individual-level estimate of the initial infection dose using the regression method and the grey band represents the 95% predictive band when the initial infection dose was estimated simultaneously with the rest of the model parameters. The reported initial infection dose is shown in red. b) The slope in a linear regression model with the natural logarithm of iRBCs per mouse as the response and the time since infection as a predictor. The red bars indicate 95% confidence intervals.

b) Graphical summary of prior and posterior distributions

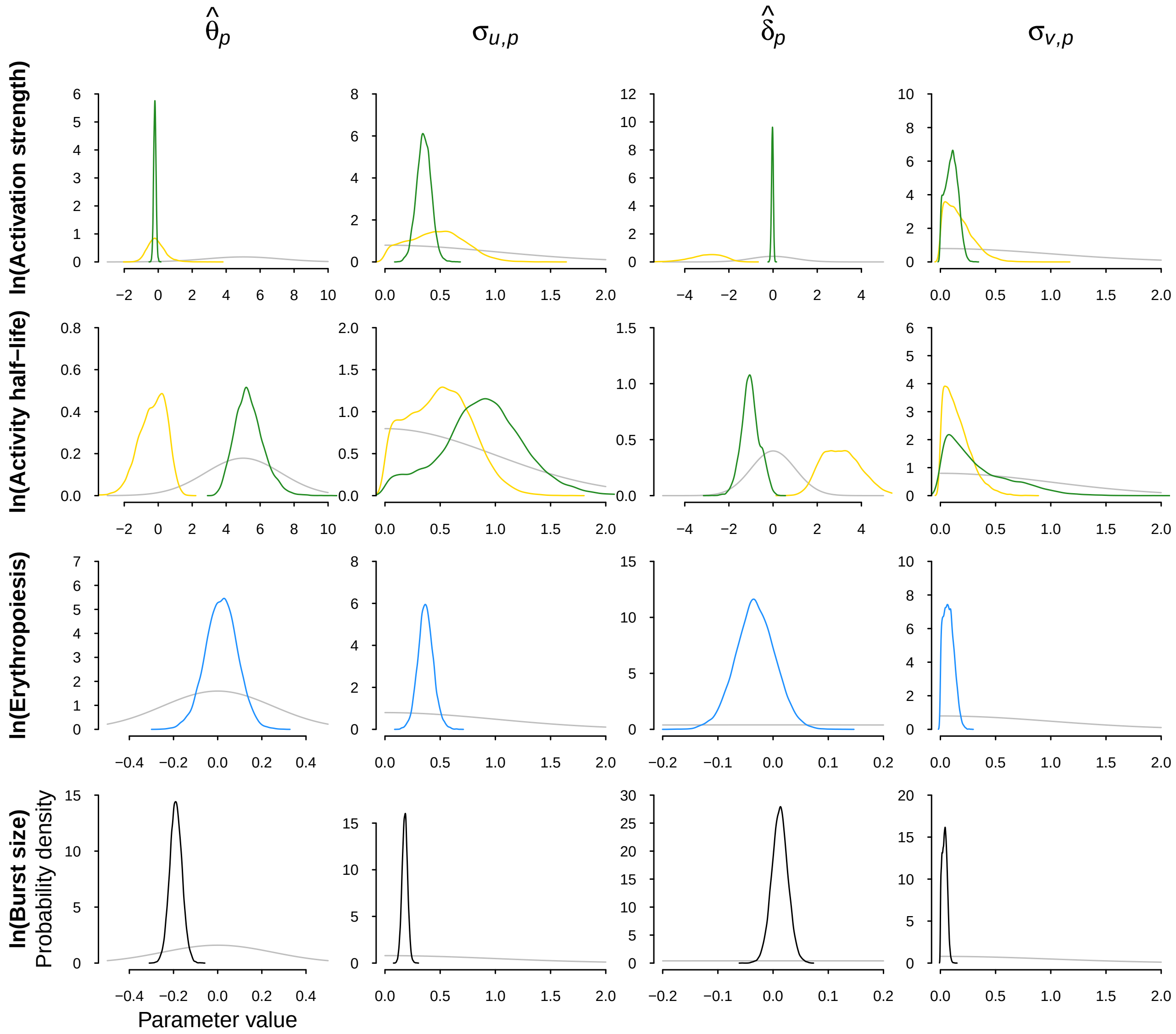

Figure B: Graphical summary of prior (grey) and posterior distributions. Erythropoiesis, general RBC clearance and targeted RBC clearance are represented by blue, yellow and green, respectively. The mathematical descriptions of prior distributions are provided in Table 1 in the main text.

c) Mice excluded from model fitting

Table A: List of mice in Timms et al. [1] excluded from model fitting and the rational for exclusion.

Dose	Block	Mouse	Rational
10^2	1	1	Infection peaks orders of magnitude lower than the rest.
10^2	1	5	Infection does not peak during the fitted time scale.
10^2	2	3	Infection never takes off.
10^3	1	5	Infection peaks five days later than the average.
10^3	2	3	Infection does not peak during the fitted time scale.
10^3	2	5	Infection never takes off.
10^4	1	1	Infection never takes off.
10^4	1	4	Infection peaks two days later than the average.
10^4	2	1	Infection peaks orders of magnitude lower than the rest.
10^4	2	2	RBC density crashes on day one for an unknown reason.
10^7	2	2	Infection peaks two days later than the average.
10^8	2	1	RBC density crashes on day one for an unknown reason.

d) Computer programmes

The computer programmes used in the present study are contained in *computer_programmes.zip.*

e) Assessment of model fit: standardised residuals

To provide a rigorous assessment of the model fit, we examined the standardised residuals for RBC and iRBC densities following Miller et al [3]. By integrating over the probability density of each parameter, Φ, the marginal standardised residual of each data point i was defined as:

$$r_{x,i} = \frac{1}{\sigma_x} \int_{\Phi} (x_{data,i} - x_{model,i}(\Phi)) d\Phi$$

where σ_x is standard deviation of x, which is either RBC or iRBC density. The fit of the full model (the parameters of which are defined by eq. 6 in the main text) to RBC and iRBC density was accurate without a significant sign of bias (Fig. C).

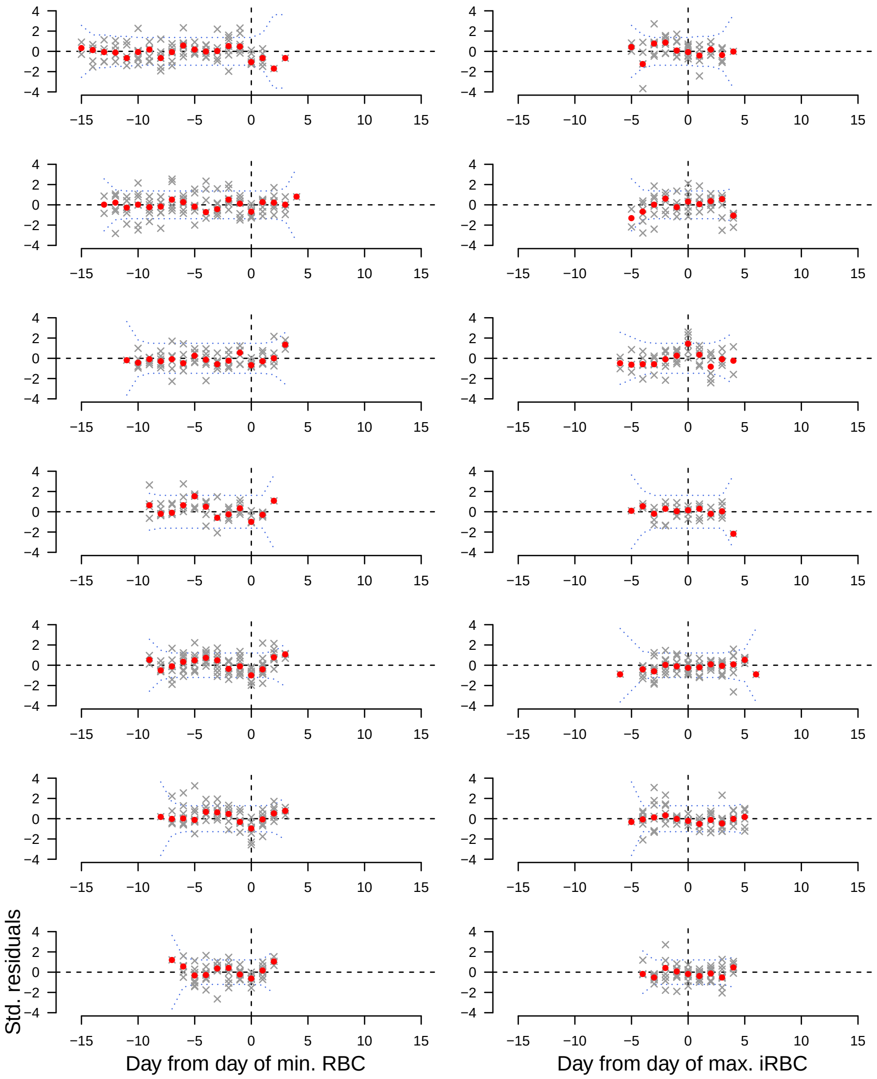

Figure C: a) Standardised model residuals of the full model (with parameters defined by eq. 6 in the main text). Poor fits are indicated by the mean residuals deviating from confidence intervals. Blue dotted lines indicated the Bonferroni-corrected 95% intervals.

f) Sensitivity of targeted iRBC clearance to dose-dependent half-life

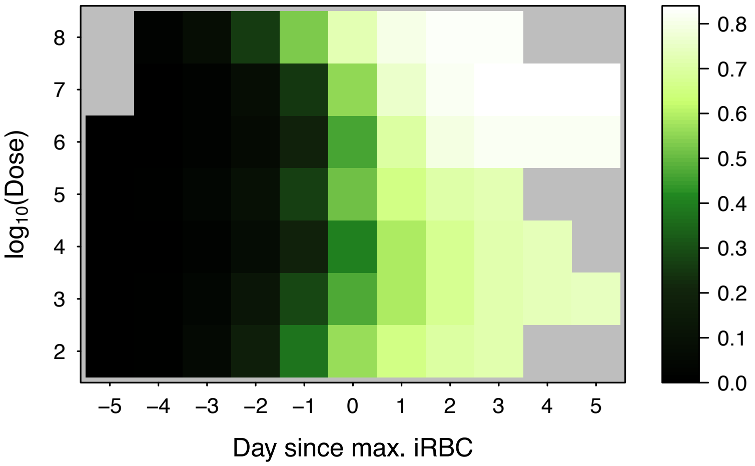

Figure D: The peak targeted iRBC clearance would increase with dose if activity half-life of this response was independent of the initial infection dose. The colour shows the median predicted targeted iRBC clearance (i.e., proportion of iRBCs cleared by the host per day) as a function of time centred around the day of peak infection (x-axis) and the initial infection dose (y-axis). The grey region indicates days beyond model fitting: our model was designed for and fitted to acute dynamics.

g) Correlations among individual variation

We estimated a correlation matrix between parameters at the individual level using an LKJ prior with a shape parameter, $\eta = 5.0$. For further details of concepts and implementation of correlation matrices in hierarchical models in Stan [4], we refer the readers to Sorensen et al. [5]. We found no moderate (> 0.3) or strong (> 0.5) correlations indicating that there is little sign of trade-offs or facilitation among parameters of host responses.

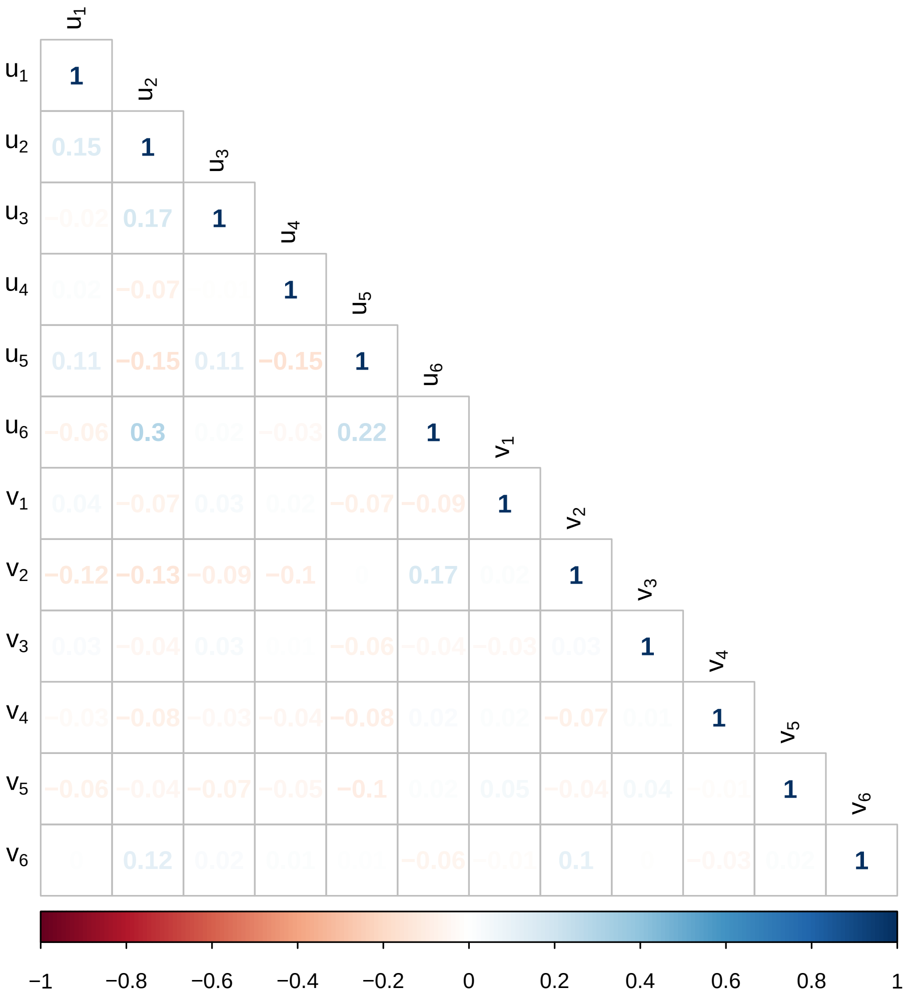

Figure E: Pairwise correlations of individual-level parameter deviations. The indexing (i.e., 1 to 6) refers to the position of the parameter set, $\theta \ni \{\psi_1, \psi_2, \phi_1, \phi_2, \rho, \beta\}$, and u and v indicate the intercept and slope variation, respectively.

References

1. Timms R, Colegrave N, Chan B, Read A. The effect of parasite dose on disease severity in the rodent malaria *Plasmodium chabaudi*. Parasitology. 2001;123(1):1–11.
2. Riches A, Sharp J, Thomas DB, Smith SV. Blood volume determination in the mouse. Journal of Physiology. 1973;228(2):279–284.
3. Miller MR, Råberg L, Read AF, Savill NJ. Quantitative analysis of immune response and erythropoiesis during rodent malarial infection. PLOS Computational Biology. 2010;6(9):e1000946.
4. Stan Development Team. RStan: the R interface to Stan, Version 2.18.2.; 2019. `http://mc-stan.org`.
5. Sorensen T, Hohenstein S, Vasishth S. Bayesian linear mixed models using Stan: A tutorial for psychologists, linguists, and cognitive scientists. Quantitative Methods for Psychology. 2016;12(3):175–200.
